# Maple Syrup, Rice and Cottonseed Meal Found to Be Adulterated*Office of Information, U. S. Department of Agriculture, Washington, D. C.

**Published:** 1913-10

**Authors:** 


### Maple Syrup, Rice and Cottonseed Meal Found to be Adulterated.*

A newly issued notice of judgment of the Department of Agriculture states that the *Vermont Maple Sugar Maker's Market, Randolph, Vt.*, pleaded guilty to the misbranding of maple syrup and was fined $50. This corporation was alleged to have shipped in violation of the Food and Drugs Act, from Vermont into the District of Columbia, a quantity of syrup labeled "Colonial Maple Syrup, prepared expressly for Woodward and Lothrop, Washington, D. C."

From analysis of a sample of the product made by the Bureau of Chemistry, it did not appear to be a high-grade syrup. It appeared that it was a syrup which had undergone fermentation, and had been reboiled, during which process it was burned. It was alleged that the product was adulterated and misbranded in that it was not maple syrup, but a by-product known as "buddy" syrup composed in part of filthy, putrid and decomposed animal or vegetable substance.

The Kellogg Manufacturing Company, Keokuk, Iowa, was fined $15 and costs for the shipment from Iowa to Illinois of rice alleged to be misbranded.

The product was labeled "This rice is finished by a coating of 1-1000th part of glucose and 1-3000th part talc, which will be removed by washing. Gate City Brand Fancy Japan Style Rice K. B. Co., Trade Mark Gate City Grown in United States, Packed by Kellogg Manufacturing Company, Keokuk, Iowa."

After examination of a sample, it was reported to be a very poor grade of domestic grown Japan style rice, coated with some preparation, presumably glucose and talc, containing a large per cent of stackburnt grains and a lot of broken grains and that it was altogether of very inferior quality.

Adulteration was alleged because a very poor grade of domestic rice had been substituted wholly or in part for the genuine article, namely, "Fancy Japan Style Rice," and further because it was coated in a manner whereby damage and inferiority were concealed. Misbranding was alleged because the product was labeled in large

*Office of Information, U. S. Department of Agriculture, Washington, D. C.

letters "Fancy Japan Style Rice" and in small inconspicuous letters "Grown in the United States" and "This rice is finished by a coating of 1-1000th part of glucose and 1-3000th part talc, which will be removed by washing."

